# Integrating Various Resources for Gene Name Normalization

**DOI:** 10.1371/journal.pone.0043558

**Published:** 2012-09-12

**Authors:** Yuncui Hu, Yanpeng Li, Hongfei Lin, Zhihao Yang, Liangxi Cheng

**Affiliations:** 1 School of Computer Science and Technology, Dalian University of Technology, Dalian, Liaoning, China; 2 Department of Biomedical Engineering, Dalian University of Technology, Dalian, Liaoning, China; Aston University, United Kingdom

## Abstract

The recognition and normalization of gene mentions in biomedical literature are crucial steps in biomedical text mining. We present a system for extracting gene names from biomedical literature and normalizing them to gene identifiers in databases. The system consists of four major components: gene name recognition, entity mapping, disambiguation and filtering. The first component is a gene name recognizer based on dictionary matching and semi-supervised learning, which utilizes the co-occurrence information of a large amount of unlabeled MEDLINE abstracts to enhance feature representation of gene named entities. In the stage of entity mapping, we combine the strategies of exact match and approximate match to establish linkage between gene names in the context and the EntrezGene database. For the gene names that map to more than one database identifiers, we develop a disambiguation method based on semantic similarity derived from the Gene Ontology and MEDLINE abstracts. To remove the noise produced in the previous steps, we design a filtering method based on the confidence scores in the dictionary used for NER. The system is able to adjust the trade-off between precision and recall based on the result of filtering. It achieves an F-measure of 83% (precision: 82.5% recall: 83.5%) on BioCreative II Gene Normalization (GN) dataset, which is comparable to the current state-of-the-art.

## Introduction

With the rapid development of biomedicine, the volume of publications indexed in MEDLINE [1], the primary database of biomedical literature, exceeds 21 million with an annual average increase rate nearly 1 million in recent years. The huge text collection brings a big challenge for experts to efficiently find useful information from it. Biomedical text mining [2] aims at making computers read and understand the large text collection as the assistants of human experts, for example, identifying the named entities of interest and extracting the interaction between proteins and genes, which would bring much benefit to biomedical research and information management.

The task of gene name normalization is to determine unique identifiers of genes and proteins mentioned in biomedical literature, so as to create the linkage between these entities and biological databases. It is closely related to the task of gene name recognition, also known as named entity recognition (NER) for gene mentions in texts, which aims to locate gene names in biomedical texts automatically and can be viewed as a prior stage of gene normalization. Both of them are important fundamental tasks in biomedical text mining and are closely related to each other.

The progress of the two tasks can be tracked by the BioCreative challenges [3]–[5], which provide an elaborately designed evaluation platform for various tasks in biomedical text mining and have attracted a number of participants both in bioinformatics and data/text mining domains. In the BioCreative I and BioCreative II challenges, there are tasks on both gene name recognition and normalization. We focus our research on the GN (gene normalization) task [6] of BioCreative II. The task is to recognize human genes in MEDLINE abstracts and normalize each gene mention to a unique identifier in the EntrezGene database. In the BioCreative II evaluation, there were 54 runs submitted by 20 participants, where the best F-measure was 81% [6] (three runs over 80%), and the median result was 73%. Lately Wermter et al. (2009) [7] reported an F-measure of 86.4%, which is the highest reported result on this dataset.

Since the methodology employed by each system involves a pipeline of different strategies, we investigate the methods from the following three stages:

1. Gene name recognition: locating gene mentions in plain texts. Some systems [8] [9] use off-the-shelf tools such as LingPipe [10] and ABNER [11] for entity recognition. The Moara Java library [12] implements a gene/protein tagger and a normalization program based on case-based reasoning. This system can be applied to various organisms flexibly, but the performance is limited by instance of training set. Machine learning (ML) based methods have shown great success by top-performing systems. Unlike dictionary-based approaches, ML-based taggers are good at generalizing to new data and are thus able to recognize gene names not seen during training.
2. Entity mapping: detecting the intention of each gene mention, and generating the candidate gene identifiers. In this stage, text strings are associated with specific gene identifiers via matching them against a lexicon. The method [13] expands the lexicon with additional resources. Although there are kinds of organism resources, such as UniProt [14] and MGI [15], it is hard to make use of these resources to generate a most comprehensive lexicon, as the identifiers and symbols from different databases are not in accordance with each other, and there is not always a direct one-to-one mapping between different database identifiers. The Peregrine system [16] analyzes the terms of the lexicon and prunes it by removing highly ambiguous terms and the terms that cause false positives.
3. Disambiguation and filtering: determining which meaning of the ambiguous gene name is in use in the context, and filtering the spurious names. When a gene mention maps to more than one identifier, disambiguation is required. CUI (Concept Unique Identifier) and MeSH features were utilized for disambiguation by Xu et al. [17]. However, they assume that among the possible gene candidates, one candidate is always the correct answer, which ignores the fact that an apparent gene mention in text may not denote a gene at all (i.e. a FP is encountered). Their results reveal that a plain bag-of-words approach performs almost equally well. ProMiner [18] heavily relies on the curation and quality of its gene dictionaries, as disambiguation is achieved by finding other synonyms of ambiguous gene names in the same text. One big challenge is that the description of gene identifiers from biological databases is usually a short text snippet which could hardly be used for constructing a good similarity function for gene disambiguation due to the lack of sufficient information.

Although great progress has been made after the challenge evaluations, we think there is still space for further improvement in each step. In particular, in gene name recognition and disambiguation stages, most of the methods use words or n-grams to represent gene names or gene description information in databases, but this method suffers from data sparseness, where low-frequency words, e.g., words in gene/protein names, cannot generate an informative classification model or similarity functions so that the performance of these methods is restricted. Addressing the problem, we develop methods to enhance the performance of gene recognition and normalization by incorporating background knowledge into the stages of gene name recognition, disambiguation and filtering. In this paper we present a system for gene name recognition and normalization, including the following steps: gene name recognition, entity mapping, disambiguation and filtering. NER method is based on our previous work [19], which utilizes the co-occurrence information of a large amount of unlabeled MEDLINE abstracts to enhance feature representation of gene named entity. The mapping method combines the strategies of exact match and approximate match implemented by an information retrieval method. For gene names that map to more than one database identifiers, we develop a disambiguation method based on semantic similarity derived from the Gene Ontology and MEDLINE abstracts. To remove the noise produced in the previous steps, we design a filtering method based on a list of terms extracted from Wikipedia and the confidence scores in the dictionary used for NER.

Our major contributions lie in:

1. We incorporate the NER system [19] into the gene normalization system, and examine its performance in the BioCreative II GN task. Most of the other work in GN tasks utilize the NER methods developed in the BioCreative I challenge, e.g., ABNER or LingPipe, which tend to be inferior to the top systems in BioCreative II, the more recent evaluation. Our system achieves an F-measure of 0.89 (the best reported result) on the BioCreative II GM test set [20], so it is interesting to see its performance in the GN task.
2. We propose the method that utilizes the confidence scores obtained by semi-supervised learning to filter the false positive predictions in the previous steps. The method makes the user able to adjust the trade-off between precision and recall of the gene normalization system, which is useful in practice and not considered in related work to the best of our knowledge.
3. Both in the stages of gene name recognition and disambiguation, we incorporate background knowledge from a large amount of MEDLINE abstracts into the system to enhance the performance, and the result shows that the huge collection of biomedical texts itself can be used as a rich resource for text mining and machine learning. Previous work [7] [17] [21] used external resources for gene name disambiguation, but our approach utilizes the unlabeled biomedical texts in different stages and integrates them in one system.

We will also compare our method with the existing work in detail in the section that introduces each component.

## Materials and Methods

**Figure 1:**
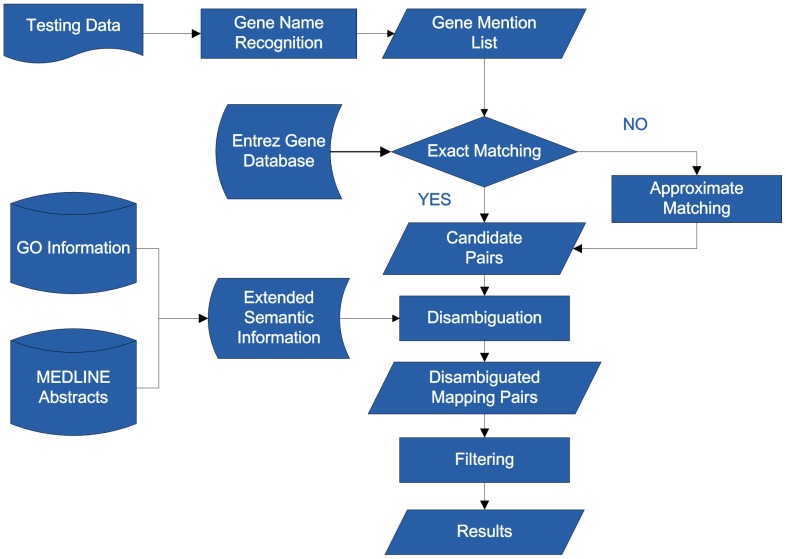
Architecture of the gene name normalization system.

Our system is composed of four components. Once gene mentions of the testing document are detected, they are mapped to gene identifiers in a synonym lexicon. Subsequently, disambiguation is implemented via an information retrieval method, which ranks the similarity scores between the context where the ambiguous gene is mentioned, and the extended semantic information of gene identifiers. In the last step, noisy items such as gene and protein family names are filtered. Figure 1 shows the overall architecture of our system.

### Gene name recognition

Gene named entity recognition aims to locate gene mentions in biomedical literature. It is a crucial preliminary step ahead of gene normalization or other text mining applications, thus attracting a lot of research interests in recent years. The result in the BioCreative II GM (gene mention) task [20] gives an overview of the current state-of-the-art, where the top-performing systems are able to achieve F-measures ranging from 0.85 to 0.87. However, these systems are not available online publicly and do not work efficiently in large datasets due to the complex combination of various high-dimensional features and sequential models [22]–[26]. The earlier well-known tools such as LingPipe [10] and ABNER [11] cannot achieve competitive results in the most recent evaluation [20].

In this work, we apply the gene mention tagger developed in our earlier work [19]. The system achieves an F-score of 0.89 on the BioCreative II GM testing set, the best reported result on this corpus. The core of the system is a dictionary constructed by feature coupling generalization (FCG), a semi-supervised learning method that generates higher-level features using the co-occurrences of raw features in huge amount of unlabeled data (17 GB unlabeled MEDLINE abstracts in our experiment). It is able to overcome the data sparseness caused by out-of-vocabulary (OOV) and extremely low-frequency words in vector space models derived from lexical feature representation. In addition, it is more efficient to run on large corpora, since the Trie tree based dictionary match [19] is faster than the ensemble of a large number of features in sequential model which are employed by other systems [20]. In addition, there is a confidence score for each dictionary entry obtained by semi-supervised learning, which can be used for filtering the noise in normalization and adjusting the trade-off of precision and recall. The detailed method will be described in the “Filtering” section.

### Entity mapping

In the gene normalization task, gene mentions found in text are required to link to their corresponding database identifier. The core of entity mapping is to measure the similarity of the gene name located in the context and the terms recorded in the biological database such as Entrez Gene. In the previous work, classical methods for similarity computation based on word overlap such as minimum edit distance [27], Dice coefficient [28], Jaro and Jaro-Winkler distance [29], and TFIDF [30] are widely used in this step and yield acceptable performance. TFIDF [31] is used in the search for dictionary entries in [31]. In [32] Lucene package is applied to index all the genes in Entrez Gene. Each mention is queried and top 50 gene IDs are returned as candidates. Here we apply a lexicon look-up based method that integrates the strategies of exact and approximate string matching.

#### Lexicon resources

The completeness of lexicon resource plays significant importance in the task of named entity recognition and gene normalization. However, it is extremely difficult to construct a dictionary that covers all the gene mentions in literature due to the huge vocabulary size of gene named entities [20]. Even carefully compiled and up-to-date gene dictionaries typically do not contain all possible synonyms or name variants. The original synonym lexicon we use is provided by the BioCreative contest, which is composed of 32975 entries, and each entry is made up of multiple synonyms indexed by the same gene identifier. In order to improve the recall of the system, for the entities not in the dictionary, we use an approximate matching method to look up the lexicon. However, this approach is likely to introduce a lot of noise that could degrade the precision of the system. So we use heuristics and threshold selection to get an appropriate trade-off between precision and recall.

#### Exact string matching

Since some variants of gene mentions are easy to be generated by simple rules without sacrificing the precision significantly, we employ the following heuristic rules to improve the coverage of the dictionary during exact matching:

1. If a space or hyphen appears in the gene mention, both the original form and the variants without the delimiter are considered. For example, for the term “NF-Kappa B”, the spelling variants including “NF Kappa B,” “NF kappa B,” “NF kappaB,” and “NFkappaB” are all put in the mapping list.
2. If an slash exists, the tokens on both sides of the oblique are considered as individual gene names and are matched against the lexicon. For example, for the term “Lef/tcf”, both “Lef” and “tcf” are matched against the lexicon.
3. The matching is case insensitive.

#### Approximate matching

As is described above, exact matching alone is not able to cover all of the gene mention variants caused by the change of word order or the removal of some very common words, e.g., “outer mitochondrial membrane translocase (34D)” -> “translocase of outer mitochondrial membrane 34.” Approximate matching approaches (such as edit distance, UnsmoothedJS, Jaro-Winkler and so on) are also used in gene normalization tasks [27]–[29] and show superior performance on recall, but the expansion of matching also brings noise and has a detrimental effect upon precision.

Addressing the problems, we employ an information retrieval based method which treats the focus gene name as a query and gene names in the lexicon as documents. When searching the dictionary, the names most similar to the query term are ranked, and the final candidates are selected from top-ranking ones. This strategy is inspired by the work [30], where TFIDF [31] is used in the search. Here we use BM25 [33] retrieval algorithm, which is reported to achieve better results than TFIDF in most IR applications, and our detailed methods for text processing and candidate selection are different from their method. The step processes are as follows: 1) each gene name in the lexicon is considered as a document, and is tokenized by splitting up non-canonical character sequences (such as punctuations, blank space, alphabetical followed by numerical characters and vice versa, etc.). 2) An inverted index is generated without consideration of stop words such as “a”, “the” and “of”. The removal of stop words is to improve the recall of the match, for example, “a PRNP gene” matches “PRNP”, where “a” may be incorrectly included in the gene name during database construction or name recognition and “gene” is a common biological term which does not distinguish different gene names. The stop list derives from two sources: the stop list [34] provided by the “RAINBOW” toolkit (http://www.cs.cmu.edu/~mccallum/bow/) containing around 500 common English words, and a list of 27 common biological terms created manually in our experiment as shown in Table 1. 3) Gene mentions found in the text are queried against this index. BM25 [33] is used to rank the candidate list.

**Table 1:** Common biological terms created manually in the stop list.

activate	family	like
anti	families	mRNA
antibody	gene	negative
cDNA	genes	promoter
complex	linked	promoters
domain	homolog	receptor
domains	homology	subfamily
dominant	human	subunit
enzymes	humans	superfamily

Each gene name in the lexicon is taken as a document *D* and each gene mention is taken as a query *Q*, containing keywords *q_1_,…,q_t_*,. The BM25 score of a document *D* and a query *Q* is computed as:
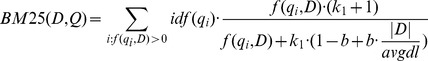
(1)
*f(q_i_, D)* is *q_i_* 's term frequency in the specific lexicon entry, |*D*| is the length of *D* in words, *avgdl* is the average length of all the lexicon entries. *k_1_* and *b* are free parameters, usually chosen as *k_1_* = 2.0 and *b* = 0.75. *idf(q_i_)* is the inverse document frequency weight of the query term *q_i_* which is defined as:
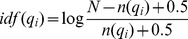
(2)


Where *n(q_i_)* is the number of gene lexicon entries containing *q_i_*, and *N* is the the lexicon size.

We consider the synonym with the highest score to be a match if the score is higher than a defined threshold. As we use Boolean queries, the actual word order within the gene mentions does not play any role. This is advantageous as word order permutations often occur when, e.g. prepositions are used. For example, the query “the human homologue of mouse Ly9” leads to full match with “ly9, mouse, homolog of” in the lexicon since “the”, “of”, “human” are removed as stop words after tokenization.

### Disambiguation

In the entity mapping stage, if multiple gene identifiers share the same gene mention, it results in ambiguity. It is necessary to pick the unique identifier for the gene mention that is most likely referred to in the text. In the work [7] [17] [21], background knowledge of a gene extracted from the EntrezGene, UniProt and GO database is employed to enhance the performance. The GNAT system [35] uses 13 types of annotations to make comparison of a gene's annotation to a text.

Each gene, as it is annotated in databases for various aspects, has a set of terms that is specific to it. Such terminology refers to where it is located on the chromosomal band, which gene family it belongs to, its species, molecular functions of its products, known mutations and single nucleotide polymorphisms, diseases caused by mutations of the gene etc., which could help to identify a gene if it is mentioned in a text. Whenever a gene (or one of its products) is discussed, some of these aspects which contain semantically relevant information will be mentioned as well. We draw hints on which gene is potentially discussed from the terminology appeared in the context.

**Figure 2:**
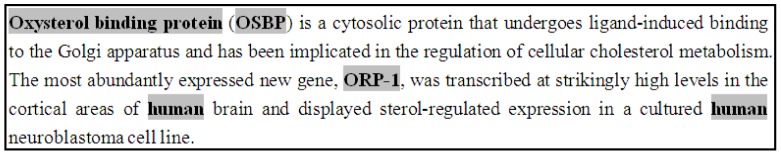
Excerpt from the abstract with PubMed ID “10588946” and partial annotation information of genes with name “ORP-1” in Entrez Gene.

The given text in Figure 2 is an excerpt from the MEDLINE abstract which ID is “10588946”. Based on the name alone, we can see that the mention “ORP-1” refers to completely different genes as shown in the annotation from EntrezGene below the text. Only comparing the gene's context to the annotation reveals that it is “oxysterol binding protein”, and the text indeed mentions “homo sapiens”. The given abstract as a whole points out that the gene “114876” out of four genes best fits the gene context.

Although a number of databases have been created to store protein information in structured and standard formats, the biomedical literature is expanding rapidly, making it difficult for database administrators to detect and manually input or update the content. Thus, most of the semantic information remains hidden within biomedical literature. To reflect the underlying sense that the genes express, we capture the reference of gene attributes from biomedical articles contained by MEDLINE database.

Our disambiguation method is based on the idea that the information associated with a gene can be a good indicator of that gene. We predict the identifier (in our case, an EntrezGene ID) with consideration of gene mention's context and gene identifier's extended semantic information. Besides short description from the EntrezGene database [36], the extended semantic information used for disambiguation is composed of manually curated GO annotations and knowledge directly derived from all MEDLINE articles known to be relevant to the gene. In EntezGene, the record of each gene contains the PMIDs of articles related to the gene, which are annotated by the database curators. We use this information to obtain the relevant articles from PubMed.

When using bag-of-word as features, general English stop words were removed. The weights of features in the vector were calculated using TF-IDF schema, which is widely used in the vector space model for information retrieval. The context vector is created using the MEDLINE abstract where the ambiguous gene symbol occurred for each testing sample. The similarity score between the context vector and relevant information vectors are calculated as the standard measurement of cosine similarity of two vectors. For the context vector *D_1_* and the relevant information vector *D_2_*, the cosine similarity between them is defined as the inner product of *D_1_* and *D_2_*, normalized by the norm of the two vectors. The formula is as below:
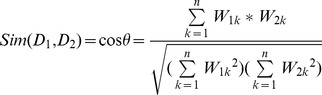
(3)where *W_1k_* and *W_2k_* are the TF-IDF values of the *k*th elements in vectors *D_1_* and *D_2_* respectively. *n* is the dimension of the vector space model. In specific, it represents the number of all terms appeared in the abstracts and in the description text.

### Filtering

In the steps of gene name recognition and entity mapping, noise of various types tends to be introduced with a negative impact on the accuracy of the system. Most methods [18] [37]–[39] relied on heuristics to remove potential false positives, including stop word lists for ambiguous or non-gene terms, and filters for nonhuman gene names. The GNAT system [35] [40] implemented a range of filters to remove likely false positive gene mentions as well as candidate IDs.

The noise can be produced in the following cases: (1) There are names which do not refer to one specific entity. Protein family names such as “CapZ Actin Capping Protein”, complexes such as “PC3” and “MCF-7”, represent a group of biological entities which have similar functions. Names like this should not be included in the final result list. (2) Approximate matching causes noise during the mapping stage. For example, the gene mention “thyroid hormone receptor” is not able to match any entry of the lexicon by exact matching. When approximate matching is used, it matches the identifier “7076”, which indexes the name “Thyroid hormone receptor-associated protein complex 230 kDa component” in the lexicon. This indicates that the established mapping is not always correct, as sometimes it maps to an identifier with a substring of some gene name. (3) In some cases, gene mentions appearing in the context serve as qualifiers, and they are not the emphasis of the statement described by authors. For example, in the MEDLINE abstract which ID is “10628838”, “furin” is annotated as a protein name. But in the sentence “…motif followed by the furin cleavage RRKKR site, a catalytic domain… ” from abstract “11255011”, “furin” is a qualifier of “cleavage RRKKR site”, thus has to be filtered.

In this work, we develop a filtering method based on the combination of the confidence scores obtained in named entity recognition, protein family names extracted from Wikipedia and the semantic similarity used in the step of disambiguation.

The whole process of the filtering method is described as bellow:

1. In the gene name recognition system, for each dictionary entry, there is a confidence score which reflects how likely the candidate is a gene name in most cases. Table 2 shows an example of the dictionary entries and confidence scores. These scores are obtained by machine learning algorithm [19], where two support vector machines (SVMs) with local lexical features and new features learned from unlabeled data are combined. In the previous system the threshold is set at −0.2, which means the entries with the score under this value are removed. However, this configuration that fitted the GM task [20] well may not perform best in the GN task, since the two tasks address different criteria for named entity recognition. Therefore, it is necessary to select the optimal setting and combine with other heuristic-based methods to achieve better performance in the GN task. In our experiment, we remove the names with the confidence score under a certain threshold from the candidate list, and examine different settings of the threshold and combine the scores with other post-processing method to enhance the performance of the GN system.
2. A list generated from the protein family names and cell lines on Wikipedia website [41] [42] is used to filter the candidate list.
3. Even if the mapping pair of gene mention and identifier from lexicon is generated, it is necessary to assess the quality on a semantic level. We retain the mapping pair only if the cosine measure from formula (3) exceeds a certain threshold. Hence, the consine measure (presented in “Disambiguation” section) has both a disambiguation function and a filtering function. The threshold is set at 0.1 in our experiments empirically.

**Table 2:** Example of the dictionary entries and confidence scores.

Dictionary entries	Confidence scores
collagen induced platelet cd62p	−0.291009
derived fibrinogen	−0.931995
dna binding inhibitory	−1.637170
gata 3 transcription factor	0.997864
lp chain	0.348884
mitochondrial ribosomal protein mrp1	0.644872
nf kb activating kinase nak	1.031284
protein hsp 60	−0.498391
peptide hormone factors	−1.072469
rel like protein binding motifs	0.174024
transforming growth factor beta family member	−0.146254

## Results and Discussion

### Experimental settings

In the experiment, we use the test set of the BioCreative II Gene Normalization taskfor evaluation, which includes a training set of 281 abstracts, a blind test set of 262 abstracts and a lexicon of 32975 distinct EntrezGene identifiers linked to a total of 163478 unique terms. The gold standard is created by expert annotators. The information of the dataset is shown in Table 3.

**Table 3:** Information of BioCreative II dataset.

Corpus	Training Set	Test Set
Abstracts	281	262
Annotations	998	1130
Entity mentions in golden answers	640	785

The evaluation metric is precision, recall and F-measure, which are defined as bellow:
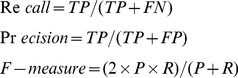
(4)where TP, FP and FN are true positive, false positive and false negative respectively. The evaluation measures are kept consistent with that in the BioCreative II challenge.

We design the following experiments to evaluate the performance of our system in several aspects. The experimental results are produced by the whole system, including the steps of gene name recognition, entity mapping, disambiguation and filtering.

**Figure 3:**
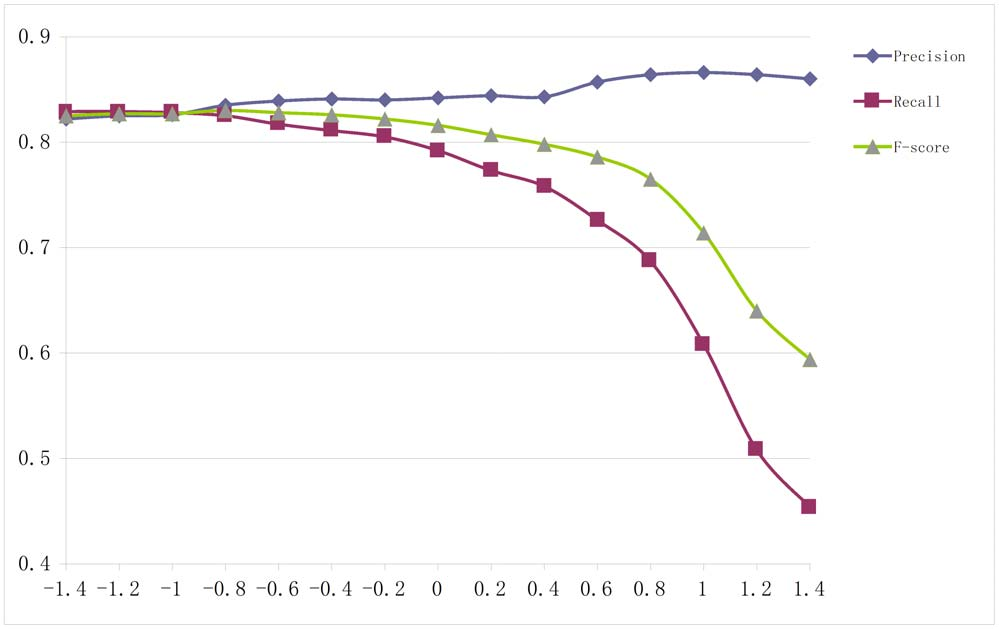
Relationship between the performance and threshold selection in filtering.

1. Comparison of different mapping and disambiguation strategies (Table 4).
2. Comparison of different filtering strategies (Table 5).
3. The relation of precision and recall when using different threshold in filtering (Figure 3). The threshold is used for filtering the names which confidence score is under the threshold. The confidence score is generated in the gene name recognition system, reflecting how likely the candidate is gene name in most cases.
4. Comparison with other systems (Table 6).

**Table 4:** Results of different mapping and disambiguation strategies.

Method	F-score	Precision	Recall	TP	FP	FN
Exact + Entrez	0.793	0.818	0.769	604	134	181
Exact + Approximate + Entrez	0.809(+2%)	0.817(−0.1%)	0.801(+4.2%)	629	141	156
Exact + Approximate + Entrez + External	0.830(+4.7%)	0.835(+2.1%)	0.825(+7.3%)	648	128	137

The first column refers to different mapping and disambiguation approaches. ‘Exact’ is short for ‘exact string matching’ and ‘Approximate’ stands for ‘Approximate string matching’; “Entrez” means using only the information in the EntrezGene database for disambiguation, and ‘External’ indicates combining external resource for disambiguation.

**Table 5:** Performance of different filtering methods.

Method	F-score	Precision	Recall
Unfiltered	0.668	0.536	0.887
List of family names and cell lines	0.733(+9.7%)	0.628(+17.2%)	0.880(−0.8%)
Semantic similarity	0.740(+10.8%)	0.667(+24.4%)	0.831(−6.3%)
Machine learning [19]	0.741(+10.9%)	0.665(+24.1%)	0.837(−5.6%)
List + Semantic similarity+ Machine learning	0.830(+24.3%)	0.835(+55.8%)	0.825(−7%)

‘unfiltered’– the method without filtering step; ‘List of family names and cell lines'– the filtering method using protein family names and cell lines extracted from Wikipedia’; ‘Semantic similarity’– the filtering method based on cosine measure calculated in the step of disambiguation; ‘Machine learning’– the filtering method based on the confidence scores obtained by machine learning in named entity recognition.

**Table 6:** Comparison with systems in the GN task of BioCreative II challenge.

Methods or authors	Precision	Recall	F-score
(Joachim et al., 2009) [7]	87.8%	85.0%	86.4%
Our System	83.5%	82.5%	83.0%
(Hakenberg et al., 2007) [21]	78.9%	83.3%	81.0%
(Fundel and Zimmer, 2007) [37]	79.2%	81.5%	80.4%
(Schuemie et al., 2007) [16]	75%	76%	75.5%
(Neves et al., 2008) [12]	55.0%	83.31%	66.26%

### Results

Table 4 shows the performance using different mapping and disambiguation approaches. In all runs of the table, the combined method of integrating all three methods introduced in the “Filtering” section is used for the step of filtering. The baseline “Exact + Entrez” uses only the information in the EntrezGene database for disambiguation, and exact string matching for mapping. It can be seen that the ‘Exact + Approximate + Entrez’ method improves the F-measure by 2%, which are caused by the improvement in recall (+4.2%). It is obvious that approximate matching can improve the recall due to the introduction of a lot of morphology variants of gene named entities. On the other hand, approximate match tends to introduce a lot of noise, but it can be seen from Table 4 that precision does not decrease, which indicates that the filtering method is effective in removing the noise caused by approximate match. In addition, the difference in performance of the second and the third runs reflects contribution of external resources. We can see that the introduction of background knowledge (‘Exact + Approximate + Entrez + External’) improves the performance by 2.6%, which shows the use of external resources is effective to disambiguation.

Table 5 compares different strategies for filtering. We can see that the strategy of filtering plays a very important role in performance improvement. The values of ‘unfiltered method’ are the performance of the system without the implementation of filtering step. It only achieves an F-measure of 66.8%, over 20% lower than the best filtering method (List+ Semantic similarity+ Machine learning), which indicates that the negative impact of noise introduced in gene name recognition and approximate match is serious. Each individual method improves the F-measure by around 10% and the combination of them leads to a further improvement. The machine learning based method for filtering achieves an F-measure of 074.1%and the performance increases to 83% when combined with other methods. One possible reason is that there are gene names not in the dictionary generated by machine learning but hit by other methods. Another is that the method based on machine learning is trained on the dataset of BioCreative II GM task [20], where the named entity concerned with is a little different from the genes defined in GN task. Therefore, the heuristic based method can be a complementary to machine learning based methods. Nevertheless, the filtering method improves the precision and F-measure by sacrificing the recall, but the overall performance (55.8% improvement in precision versus 7% decrease in recall) is at an acceptable level.

Another advantage of the filtering method is that it provides flexibility for users to adjust the trade-off between precision and recall, which is very useful in practice. For example, if the user needs a system with high precision, he can increase the threshold to get an expectable precision. Figure 3 shows the impact of threshold selection, where we can see that generally the precision increases and the recall decreases with the threshold increasing. The best F-measure is 83%, where the precision is 83.5%, the recall is 82.5% and the threshold is −0.8.

In addition, to examine the robustness of the method for gene normalization, we also took the gene mentions from the gold standard as testing data. All the steps of normalization were implemented on the gene mention list from the gold standard. We got an F-value of 90.5% (precision: 94.3% recall: 87.0%). This result suffered no influence from the name recognition system. It can be seen that the overall performance of our method is stable and will not be affected by the difference coming from preprocessing.

In Table 6, we compare the performance of our system with some top results of official runs in the BioCreative II GN task and some post-submission work [7]. It shows that our method is comparable to the current state-of-the-art in this task. From both Table 4 and 5, we can see that the combination of the filtering method and background information based disambiguation method have the major contribution to improve the performance.

The performance of our system is not as good as the GeNo system [7]. However, we made use of a large amount of unlabeled biomedical texts in the stages of gene name recognition, disambiguation and filtering. And the incorporation of this resource enhanced our results.

### Error analysis

We analyzed the FN and FP errors for the best-performing scenario in Table 4, and categorized the causes of errors as shown in Table 7 and 8.

**Table 7:** Causes for FN errors in the normalization task.

FN causes type	Frequency	Proportion
Mentions not identified	56	40.9%
Lexicon deficiencies	31	22.6%
Incorrect disambiguation	28	20.4%
Erroneously filtered	22	16.1%

**Table 8:** Causes for FP errors in the normalization task.

FP causes type	Frequency	Proportion
Spurious gene names	95	74.2%
Noise caused by searching	7	5.5%
Incorrect disambiguation	26	20.3%

56 of the FN (40.9%) are caused by gene mention detection system. More specifically, enumeration such as “cofactors A, D, E, and C” and “ORP-1 to ORP-6” cannot be identified. Because of the scale of lexicon, 31 gene mentions cannot get matched. Despite extended semantic information is employed for disambiguation, still 28 of the FN (20.4%) and 26 of the FP (20.3%) errors are due to the disambiguation as similarity of the different senses is so high that it is hard to distinguish between them. For example, the mention “CBF-B” (Core-binding factor subunit beta) in abstract 15243141 can map to 3 identifiers in the lexicon which are “4800”, “4801” and “865”. These senses share a considerable amount of common information.

Our method computed the similarity for each pair and finally associated it with “865”, while the golden standard is “4801”. Although we have used the extended information to disambiguate, in some cases our system can not distinguish among these symbols according to the context. The filtering component is responsible for 22 of the FN errors (16.1%) by erroneously filtering the TPs as their semantic similarity score is lower than the threshold.

95 FPs (74.2%) results from spurious gene names which appear as gene names but do not refer to gene in the current context, such as the name “furin” mentioned in section 3.4, behaving as TP in one abstract while as FP in another. Also, the list of protein families and complexes generated from Wikipedia is not complete. 7 of the FPs (5.5%) are due to the noise caused by searching during the mapping. For example, the mention “rho family GTPases” appeared in abstract 11084341 was assigned to gene “23580”, which refers to “Binder of Rho GTPase 4”, but this result is an incorrect mapping. This could be due to the BM25 ranking, and it could also be caused by the disambiguation step.

### Conclusions and future work

In this paper, we present a system for gene name recognition and normalization in biomedical literature. The performance is comparable to the state-of-the-art in the BioCreative II GN dataset. It is interesting to note that in the steps of gene name recognition, disambiguation and filtering, unlabeled MEDLINE abstracts are incorporated as background knowledge to enrich the representation of gene name and the description information in databases, and these methods improve the performance of the final system. We think there is space for further improvement if we make better use of the rich background knowledge. For example, we may generate a larger gene name dictionary using the combination of term classification and bootstrapping so that the recall can be further improved. Also we can learn an enriched representation for both context and database identifiers using semi-supervised learning strategies. In addition, inspired by the error analysis, we may consider some heuristic methods for post-processing, for example, identifying the enumeration and compound biomedical names.
